# Identification of a novel downstream *single-minded* midline regulatory element in *Drosophila melanogaster*

**DOI:** 10.17912/micropub.biology.001317

**Published:** 2024-10-17

**Authors:** Geyenna Sterling-Lentsch, Marc S. Halfon

**Affiliations:** 1 Department of Biochemistry, University at Buffalo, State University of New York, Buffalo, New York, United States

## Abstract

Development of the *Drosophila melanogaster* central nervous system midline depends on the gene *single-minded* ( *sim* ). Although *sim* regulation has been studied extensively, the fact that an enhancer mediating late embryonic *sim* transcription has not been identified suggests that additional regulatory sequences remain unknown. We tested several evolutionarily conserved sequences in the *sim* downstream region and isolated *sim_3pB* , whose midline activity in a reporter gene assay begins later than previously characterized *sim* enhancers. Its activity shares several key similarities with the *Aedes aegypti sim* _ *5P3* enhancer, though is sufficiently different to warrant further investigation into how *sim_3pB* functions in its native context.

**Figure 1. Characterization of putative midline enhancers in the sim 3’ region:**
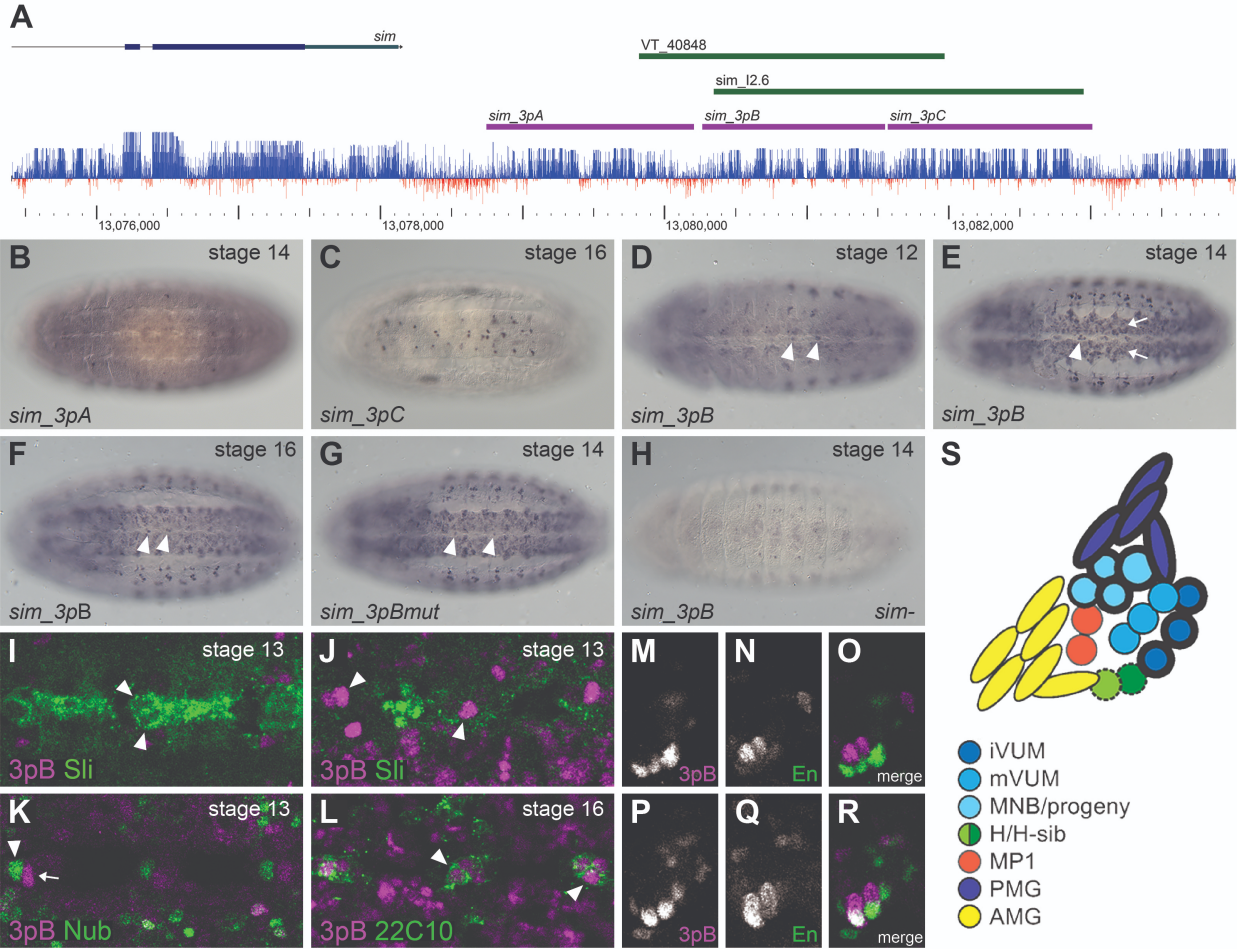
(A) Schematic of the *D. melanogaster* *sim* downstream region. Genomic sequences chosen for testing are colored magenta; previously published regions overlapping *sim_3pB* are green. PhyloP sequence conservation across 27 insects lies at the bottom, with conserved sequence shown in blue and areas of low conservation shown in red. (B) The *sim_3pA* fragment fails to drive reporter expression in transgenic *D. melanogaster* embryos. Shown at stage 14. (C) Reporter expression driven by *sim_3pC* at stage 16 in the CNS, outside of the midline. (D-F) Reporter expression driven by *sim_3pB* at stages 12 (D), 14 (E), and 16 (F) in the CNS and other tissues. Midline expression begins at the end of stage 12 and continues to at least stage 16, with a peak in stages 13 and 14. Arrowheads show midline staining. Arrows in (E) point to ectopic CNS staining. (G) Some *sim_3pB* activity persists with mutation of CME sites; midline activity is reduced while background expression is mostly unchanged. Stage 14 is shown. Arrowheads show positive midline cells. (H) *sim_3pB* activity in the entire CNS is essentially absent in a *sim* null environment; stage 14 is shown. Embryos in B-H are all oriented ventral side up with anterior to the left. (I-J) Co-labelling of the *sim_3pB* reporter (3pB; magenta) and Slit (Sli; green) shows no overlap with PMG or AMG at stage 13 (ventral view; arrowheads point to midline cells). (K) Co-labelling of *sim_3pB* (3pB; magenta) and Nubbin (Nub; green) shows no overlap with MP1 neurons or H-cell/H-cell sib at stage 13 (ventral view; arrow and arrowhead point to a *sim_3pB* positive cell and nubbin positive cell, respectively). (L) Co-localization of *sim_3pB* (3pB; magenta) and 22C10 (green) suggests *sim_3pB* expression in the mVUMs at stage 16 (ventral view; arrowheads point to midline cells with colocalized marker). (M-R) Sagittal views of midline segments co-stained for *sim_3pB* and Engrailed in stage 13 embryos. (M-O) *sim_3pB* is active in the mVUMs, which are not Engrailed-positive. (P-R) *sim_3pB* sporadically overlaps Engrailed expression in the iVUMs. (S) Cartoon of a midline segment at stage 13, sagittal view (adapted from (Zhang et al., 2011)). Cells with heavy borders express Engrailed.

## Description

In Arthropods, development of the central nervous system relies heavily on the gene *single-minded* ( *sim* ; FlyBaseID: FBgn0004666) (Linne et al., 2012) . *sim* encodes a bHLH-PAS transcription factor that acts as a key regulator in the development of the ventral midline (Crews et al., 1988; Nambu et al., 1991) . In the fruit fly *Drosophila melanogaster* , *sim* is active throughout the whole of embryogenesis, with transcript first being detected at the cellular blastoderm stage and persisting until the end of embryo development (Thomas et al., 1988; Wheeler et al., 2006) . Most of the noncoding DNA of the *sim* locus has been assayed for regulatory activity, and enhancers have been defined for early embryonic, larval, and adult stages of development (Freer et al., 2011; Hong et al., 2013; Jenett et al., 2012; Kvon et al., 2014; Markstein et al., 2004; Pearson et al., 2016; Sandmann et al., 2007) . However, *sim* regulation is still not completely defined. For example, enhancers governing midline expression later in embryonic development have yet to be identified. Although much of the locus has already been characterized, we reasoned that a finer-scale mapping of specific regions might better resolve discrete regulatory activities and possibly discover unknown enhancers, improving our understanding of *sim* regulation.

In this study, we focus on the 3' end of *D. melanogaster sim* , where sequence comparison across 27 insects shows distinct blocks of high conservation ( Fig. 1A ). Using this conservation as a guide, we isolated three fragments— *sim_3pA* , *sim_3pB* , and *sim_3pC* —from the genome and characterized them by reporter gene assay in transgenic embryos. *sim_3pA* is negative in all embryonic tissues at all stages ( Fig. 1B ). *sim_3pC* shows limited activity in the central nervous system (CNS) of stage 16 embryos, but this expression is outside of the midline in regions ectopic to *sim* ( Fig. 1C ). Therefore, this element is likely not a true *sim* enhancer. Neither *sim_3pC* nor *sim_3pA* were explored further. *sim_3pB* , on the other hand, is active in the midline from the end of germband retraction (late stage 12) through at least stage 16, with the strongest signal at stages 13 and 14 ( Fig. 1D -F, arrowheads). *sim_3pB* also displays extensive ectopic activity throughout the medial portion of the CNS, starting earlier at approximately stage 10 and continuing into stage 16 ( Fig. 1E, arrows).

All known *sim* midline enhancers in *D. melanogaster* possess autoregulatory capability mediated by binding of Sim:Tango heterodimers to CNS Midline Elements (CMEs) (Pearson and Crews, 2014; Sonnenfeld et al., 1997; Wharton et al., 1994) . Three matches to the CME consensus sequence, RCGTG (Hong et al., 2013; Sonnenfeld et al., 1997) , are present in *sim_3pB* . However, mutation of these sites leads to only a modest reduction in reporter expression. Midline activity is slightly reduced, whereas background expression is mostly unchanged ( Fig. 1G, arrowheads). Careful quantitative analysis will be required to confirm whether this difference in activity is significant. Surprisingly, when introduced into a *sim* null environment, *sim_3pB* activity is strongly reduced in the entire CNS ( Fig. 1H ). These contradictory results suggest that *sim_3pB* may be both directly and indirectly dependent on Sim for activity, although we cannot rule out additional direct Sim autoregulatory binding through non-canonical sites.

During early stage 11 in *D. melanogaster* , the embryonic midline consists of the anterior and posterior midline glia (AMG and PMG, respectively), the median neuroblast (MNB), and midline precursors (MP1, MP3, MP4, MP5, and MP6) (Wheeler et al., 2006) . Toward the end of stage 11, the MP3 precursors divide into H-cell and H-cell sib while MP4-6 each give rise to one Ventral Unpaired Median motoneuron and one Ventral Unpaired Median interneuron (mVUM and iVUM, respectively). Each cell type expresses a specific set of genes that allows it to be uniquely identified (Wheeler et al., 2006) . By co-staining *sim_3pB* embryos for the reporter gene and one of four known midline markers—Slit, Mab 22C10, Engrailed, and Nubbin—we were able to identify the midline cells in which *sim_3pB* is active. In stage 13/14 embryos, *sim_3pB* activity did not coincide with either Slit or Nubbin, which rules out midline glia (AMG/PMG), MP1 neurons, and H-cell/H-cell sib ( Fig. 1I -K). Co-localization with 22C10 in stage 16 embryos suggests activity in the mVUMs ( Fig. 1L ), which is confirmed by *sim_3pB* staining relative to Engrailed-positive cells at stage 13 ( Fig. 1M -O, S). Additionally, sporadic activity is observed in the iVUMs ( Fig. 1P -R, S). *sim* itself is not expressed in the mVUMs at stage 13 (Wheeler et al., 2006) , but is it possible that activity begins weakly prior to stage 13 (in the MP4-6 precursor cells) and is already fading by the time they have divided. This could also explain the inconsistent activity seen in the iVUMs.

*sim_3pB* shares several characteristics with a recently discovered *sim* midline enhancer in the mosquito *Aedes aegypti* , *sim_5P3* (Schember et al., 2024) . It is also dissimilar in key ways. Although both elements begin activity later than other known *sim* midline primordium enhancers, *sim_5P3* becomes active at the end of stage 9 and *sim_3pB* not until the end of stage 12 (with respect to its midline activity). Neither are active in the full set of midline cells and are instead restricted to the MP4 lineage, but whereas *sim_5P3* expression includes all the descendants of the initial MP4 equivalence group (PMG, MNB, the MP4-6 precursors and their VUM progeny), *sim_3pB* appears limited to the VUMs. Additionally, while neither element is directly autoregulated, they differ in their dependance on Sim protein. The *sim_5P3* sequence contains no CME sites and is fully functional in a *sim* null background. On the other hand, *sim_3pB* remains weakly active with the loss of three CME sites yet is not functional in a *sim* null background. Taken together, despite interesting similarities between these two regulatory elements, it cannot necessarily be concluded that *sim_3pB* is a functional counterpart to *A. aegypti* *sim_5P3* .

*sim_3pB* activity, though it includes *sim* positive cells, also extends to regions where *sim* is not expressed in the embryo. The sequence we isolated could be lacking repressors necessary for midline-only activity. Alternatively, *sim_3pB* may not act as an enhancer in its native context. Kalay et al. (2019) introduced the concept of “cryptic enhancers”—sequences that have the ability to drive gene expression in isolation but are repressed in their endogenous chromosomal environment. A cryptic enhancer might thus be located within a larger region that is negative for regulatory activity (Kalay et al., 2019) . Such may be the case with *sim_3pB* . In two separate experiments, regions containing and extending beyond the *sim_3pB* sequence failed to drive reporter expression: *sim_I2.6* , which overlaps all but the first ~70bp of *sim_3pB* (Freer et al., 2011) , and *VT40848* , which contains the entirety of the *sim_3pB* sequence (Kvon et al., 2014) , were both reported to be inactive ( Fig. 1A ). Taking this into consideration, it is possible that *sim_3pB* is a cryptic enhancer for *sim* . An intriguing alternative possibility is that *sim_3pB* acts in its native location as a silencer element to prevent *sim* transcription in the mVUMs and cells outside of the midline, but manifests as an enhancer in an out-of-context reporter assay. This could explain the extensive activity observed in *sim-* negative cells. Further investigation will be necessary to test these various possibilities. Nonetheless, currently *sim_3pB* stands as the only identified embryonic *D. melanogaster* *sim* regulatory element with midline activity beginning at or after the late midline primordium stage. Continuing to explore the *sim* locus may reveal more enhancers governing midline activity at these later stages, providing a clearer picture of how *sim* is regulated throughout embryonic development in *D. melanogaster* and in Arthropods as a whole.

## Methods

*Reporter Constructs*

*sim_3pA* and *sim_3pC* were isolated via PCR on genomic DNA from *D. melanogaster* strain OregonR (OrgR) and cloned into the *pattBnucGFP* vector (Kazemian et al., 2011) . *sim_3pB* was synthesized as a gBlock (IDT, Coralville IA) and cloned into the *placZattB* vector (Bischof et al., 2013) using the NEBuilder HiFi DNA Assembly kit (New England Biolabs, Beverley, MA). To generate the *sim_3pBmut* line, MacVector (Apex, NC) was used to scan for Sim:Tgo binding sequences ACGTG (Sonnenfeld et al., 1997) and DDRCGTG (Hong et al., 2013) . Three matches were found, and each site was mutated to GGATCC, as described by Wharton et al. (1994). The *sim_3pBmut* fragment was synthesized as a gBlock (IDT, Coralville IA) and inserted into *placZattB* using the NEBuilder HiFi DNA Assembly kit. Transgenic flies were generated by Rainbow Transgenic Flies (Camarillo, CA) by phiC31 recombination using line attP2 8622.

*Sequence conservation*

Sequence conservation was obtained from the UCSC Genome Browser (Raney et al., 2024) using the “phyloP 27 species” conservation track. Data in this track are obtained using phyloP (Pollard et al., 2010) and the following species: *Drosophila melanogaster, D. simulans, D. sechellia, D. yakuba, D. erecta, D. biarmipes, D. suzukii, D. ananassae, D. bipectinata, D. eugracilis, D. elegans, D. kikkawai, D. takahashii, D. rhopaloa, D. ficusphila, D. pseudoobscura, D. persimilis, D. miranda, D. willistoni, D. virilis, D. mojavensis, D. albomicans, D. grimshawi, Musca domestica, Anopheles gambiae, Apis mellifera, Tribolium castaneum.*

*Immunohistochemistry*

Immunohistochemistry on *D. melanogaster* embryos was conducted using standard methods. Staining was done with primary antibodies mouse anti-β-galactosidase (1:500, Promega Z378) and rabbit anti-GFP (1:10000, Ab-cam ab290) then visualized using the Vectastain® Standard ABC kit (Vector Laboratories). Imaging was performed using a Zeiss Axioskop equipped with a Jenoptix Arctur camera. For fluorescent staining, primary antibodies were mouse anti-Slit C555.6D (1:10; Developmental Studies Hybridoma Bank), mouse anti-Nubbin 2D4 (1:10, DSHB), mouse anti-Engrailed 4D9 (DSHB), and Mab 22C10 (1:20, DSHB). Secondary antibodies were anti-mouse Alexa Fluor 488 (1:250, Molecular Probes, A11029) and anti-rabbit Alexa Fluor 647 (1:500, Molecular Probes, A21245). Fluorescent staining was visualized using a Leica SP8 confocal microscope.

*Test for autoregulation*

The *sim_3pB* construct was recombined onto a *sim* null chromosome ( *sim ^2^* , Bloomington Stock Center stock #2055) and balanced with a *TM3* balancer containing *ftz-lacZ* . Loss of *sim* function was confirmed by complementation testing with a *sim-* null deficiency ( *Df(3R)Excel6167/TM6B,* Bloomington Stock Center stock #7646).

## Reagents

**Table:** 

**Plasmid**	**Features**	**Description**	**Available from**
*pattBnucGFPh*	white[+] SV40 3'UTR nucEGFP gene hsp70 minimal promoter MCS attB ampR	ϕC31-enabled vector containing *eGFP* under control of a minimal hsp70 promoter. Polylinker and eGFP cassette from pH-Stinger.	Authors, on request
*pLacZattB*	white[+] SV40 3'UTR lacZ gene hsp70 minimal promoter MCS attB loxP ampR	ϕC31-enabled vector containing *LacZ* under control of a minimal hsp70 promoter.	Drosophila Genomics Resource Center; stock 1421

**Table:** 

**Line**	**Genotype**	**Available from**
a *ttP2 8622*	P{CaryP}attP2	Bloomington Drosophila Stock center; stock #8622
*sim ^2^*	sim[2] kar[1]/TM3, P{ry[+t7.2]=ftz-lacC}SC1, Sb[1] ry[RK]	Bloomington Drosophila Stock center; stock #2055
( *Df(3R)Excel6167/TM6B*	w[1118]; Df(3R)Exel6167, P{w[+mC]=XP-U}Exel6167/TM6B, Tb[1]	Bloomington Drosophila Stock center; stock #7646
*Dr/TFLZ*	TM3, P{ry[+t7.2]=ftz-lacZ.ry[+]}TM3, Sb[1] ry[*]/Dr[Mio]	Bloomington Drosophila Stock Center; stock #3218

**Table:** 

**Antibody**	**Animal and clonality**	**Description**	**Available from**
Mab 22C10	Mouse; monoclonal	Raised against full-length *Drosophila melanogaster* Futsch sequence	Developmental Studies Hybridoma Bank
anti-Engrailed 4D9	Mouse; monoclonal	Antibody recognizes both the Engrailed and Invected gene products of *Drosophila* .	Developmental Studies Hybridoma Bank
anti-Nubbin 2D4	Mouse; monoclonal	Raised against recombinant Nub/Pdm sequence from *Artermia*	Developmental Studies Hybridoma Bank
anti-Slit C555.6D	Mouse; monoclonal	Raised against recombinant fusion protein containing the C-terminal region of *Drosophila* Slit, aa 1311-1480 (isoform A; Uniprot ID P24014-2)	Developmental Studies Hybridoma Bank
anti-GFP ab290	Rabbit; polyclonal		Ab-cam
anti-β-galactosidase	Mouse; monoclonal		Promega #Z378
anti-mouse Alexa Fluor 488	Goat; polyclonal		Molecular Probes #A11029
anti-rabbit Alexa Fluor 647	Goat; polyclonal		Molecular Probes #A21245
anti-mouse IgG (biotinylated)	Goat; polyclonal		Vector Laboratories
anti-rabbit IgG (biotinylated)	Goat; polyclonal		Vector Laboratories
